# *De novo* Assembly and Annotation of the Antarctic Alga *Prasiola crispa* Transcriptome

**DOI:** 10.3389/fmolb.2017.00089

**Published:** 2018-01-08

**Authors:** Evelise L. Carvalho, Lucas F. Maciel, Pablo E. Macedo, Filipe Z. Dezordi, Maria E. T. Abreu, Filipe de Carvalho Victória, Antônio B. Pereira, Juliano T. Boldo, Gabriel da Luz Wallau, Paulo M. Pinto

**Affiliations:** ^1^Applied Proteomics Laboratory, Federal University of Pampa, São Gabriel, Brazil; ^2^Núcleo de Estudos da Vegetação Antártica, Federal University of Pampa, São Gabriel, Brazil; ^3^Department of Entomology, Aggeu Magalhães Institute (IAM), Recife, Brazil

**Keywords:** RNA-seq, Trebouxiophyceae, Prasiolales, transcriptome, extreme environments, anti-freeze proteins

## Introduction

The Antarctic, located on the South Pole of the Earth and isolated from other continents by the Atlantic, Pacific and Indian oceans. It is considered a continent with severe environmental conditions for the development of life, thus limiting the Antarctic fauna and flora to specific organisms that have survival adaptation mechanisms (Jackson and Seppelt, 1997). The average annual precipitation of Antarctic is only 200 mm with winds of 327 km/h and temperatures below −90°C have already been recorded (Martínez-Rosales et al., 2012). The total area is 14,000,000 km2, with 98–99.7% covered by snow and ice, with layers averaging 1.6 km thick (Convey et al., 2008; Martínez-Rosales et al., 2012). In addition, the ozone hole over the Antarctic region, first described in the 1980s, causes a high rate of ultraviolet radiation over the region, which is intensified by the ice-generated reflection (Kuttippurath and Nair, 2017; Marizcurrena et al., 2017).

Among the algae present on the Antarctic ice-free areas, *Prasiola crispa* (Lightfoot) Kützing is the most commonly found organism. *P. crispa* is a green macroalga belonging to the Trebouxiophyceae class and is among the most important primary producers in the Antarctic territory. *P. crispa* occurs in hydro-terrestrial soils, in the supralittoral zones of the maritime and continental Antarctica, where they form large and green carpets on the humid soil. *P. crispa* is found close to bird populations, mainly adjacent to penguin colonies, where the soil is rich in guano, a substrate with a high incidence of uric acid and nitrogen compounds (Kováčik and Pereira, 2001).

The morphology of these algae varies from unisserated filaments to stalks in the form of a tape, expanded blades or packages as colonies, which are characterized by a large phenotypic plasticity related to environmental factors (Rindi et al., 2007).

During the course of the seasons, *P. crispa* needs to tolerate extreme environments, such as repeated freeze and thaw cycles, physiological drought, salinity stress, and high levels of UV radiation (Jacob et al., 1992; Jackson and Seppelt, 1997). However, the genes associated with these adaptive characteristics in *P. crispa* remain unknown. Therefore, to better understand the genetic and metabolic adaptations that allow this organism to survive in harsh environments, we sequenced its transcriptome.

Transcriptomes represent all the expressed fractions of genomes and are a viable alternative to understand and characterize genome wide genetic information of organisms since it simplifies genetic analyses, as compared to whole genome sequencing (Riesgo et al., 2012).

High-throughput sequencing of transcriptomes (RNA-Seq) has provided new routes to study the genetic and functional information stored within any organism at an unprecedented scale and speed. Transcriptome approaches have been employed in a large number of the studies involving non-model organisms, which normally lack reference genomes (Ekblom and Galindo, 2011; Haas et al., 2013).

Among these organisms are the algae group. The available data consists of organisms belonging to different phylum, such as Prymnesiophyte (Koid et al., 2014), Chlorophyta (Rismani-Yazdi et al., 2011), Haptophyta (Talarski et al., 2016), Stramenopiles (Im et al., 2015), and Rhodophyta (Shuangxiu et al., 2014).

*P. crispa* represents the first organism of the Prasiolales order with an available transcriptome since, until this work, the mitochondrial and plastid genomes were the only molecular data available for this species (Carvalho et al., 2015, 2017). Therefore, the purpose of this study was to sequence the transcriptome of *P. crispa*. The identification of transcripts will help to identify genes that are responsible for organism survival in this environment, as well as assisting in future genetic, phylogenetic, and biotechnological studies of *P. crispa* and other Antarctic organisms.

## Experimental design, materials, and methods

### Algae collection

*P. crispa* was collected in areas near the Arctowski Polish Station Region, Admiralty Bay, King George Island (61°50′−62°15′ S and 57°30′−59°00′W), Antartic. The collection was carried out in the Antarctic summer, in January of 2014 Austral summer, with temperature ranging from 0.5 to 2.0°C. The samples were maintained in RNAlater® (Sigma-Aldrich, USA) until RNA extraction.

### Total RNA extraction and RNA-Seq

Total RNA was extracted from three pools of samples using an RNAqueous®-Micro Total RNA Isolation Kit (Thermo Fisher Scientific Inc., USA) according to the manufacturer's instructions. The RNA-Seq library was prepared using random primers. The transcriptome was sequenced by Macrogen Service using the Solexa-Illumina HiSeq 2000 next-generation sequencing platform device according to the manufacturer's instructions. A paired-end reads with a read size of ~100 bp separated by insert size of 300 bp was employed.

The BioProject ID of our data is PRJNA329112, and the BioSample accession number is SAMN05392062. All raw reads were deposited into the Sequencing Read Archive (SRA) of NCBI with accession number SRR5754271. This Transcriptome Shotgun Assembly project has been deposited at DDBJ/EMBL/GenBank under the accession GFTS00000000.

### *De novo* transcriptome assembly

Raw reads from data sets were filtered to remove the adapter sequences, and low quality reads with Fastx-toolkit (quality cut-off = 30) (Gordon and Hannon, 2010) and Trimmomatic v 0.36 using default parameters (Bolger et al., 2014). Next, we used Trinity version r2014-07-17 (Ekblom and Galindo, 2011) as Bruijn graph assembler with 25 kmer size. Due to the sequencing of a complex sample extracted from the Antarctic soil, we expected some amount of bacterial and fungal contamination. Therefore, in order to remove such contaminants, we performed a tblastx with default parameters (Altschul et al., 1990) searching against all of the NCBI nucleotide non-redundant database and recovered all contigs in which the best blast hit occurred with algae and plant sequences. Next, we used Bowtie2 (Langmead and Salzberg, 2012) with default parameters to recover only the reads that mapped against those *P. crispa* contigs.

### Functional annotation

The assembled and recovered contigs were searched against the NCBI protein non-redundant database using the BLASTX algorithm; the *E*-value cut-off was set at 1e-10. Genes were tentatively identified based on the best hits against known sequences. Blast2GO (Conesa and Götz, 2008) was used for mapping and annotation, associating Gene Ontology (GO) terms and predicting their function. Assembled and annotated transcriptome is publicly available on Figshare at: https://figshare.com/s/a60ae8a0445d547b9360.

After a stringent filtering process, the processed reads were assembled into 17,201 contigs. Statistics of the assembly are summarized in Table 1. CD-HIT-EST version 4.6.8, 2017-06-21 (Fu et al., 2012) was used for clustering of assembled transcripts with the default parameters with sequence identity threshold set to 95%, in order to indicate the number of unigenes. The clustering reduced the number of transcripts marginally by 3.5%. Our analysis showed that 93% of the unigenes are represented by only one isoform. The metrics of *P. crispa* were compared with others transcriptomes of the organism from the Trebouxiophyceae class, including *Chlorella minutissima* (Yu et al., 2016), *Trebouxia gelatinosa* (Carniel et al., 2016), *Coccomyxa subellipsoidea* (Peng et al., 2016), *Chlorella sorokiniana* (Li et al., 2016), *Botryococcus braunii* (Xu et al., 2015), and was found to have the lowest number of total reads. In relation to the number of contigs, *P. crispa* is in an intermediary position, with *C. sorokiniana* being the organism with the highest number of contigs (63,811) and *C. subellipsoidea* having the lowest number of contigs (9,409). More information on the metrics of transcriptomes is given in Supplementary Table S1.

**Table 1:** Summary of *Prasiola crispa* assembly.

**Attributes**	**Value**
Total raw reads	42,978,976
Total processed reads	5,233,428
Number of contigs	17,201
Total length (pb)	13,127,645
N50	1,036
GC (%)	49.66
Average size (pb)	763.19
Size range (pb)	200–12,802
Contigs > 1 kb	3,973 (22.05%)

**Figure 1:**
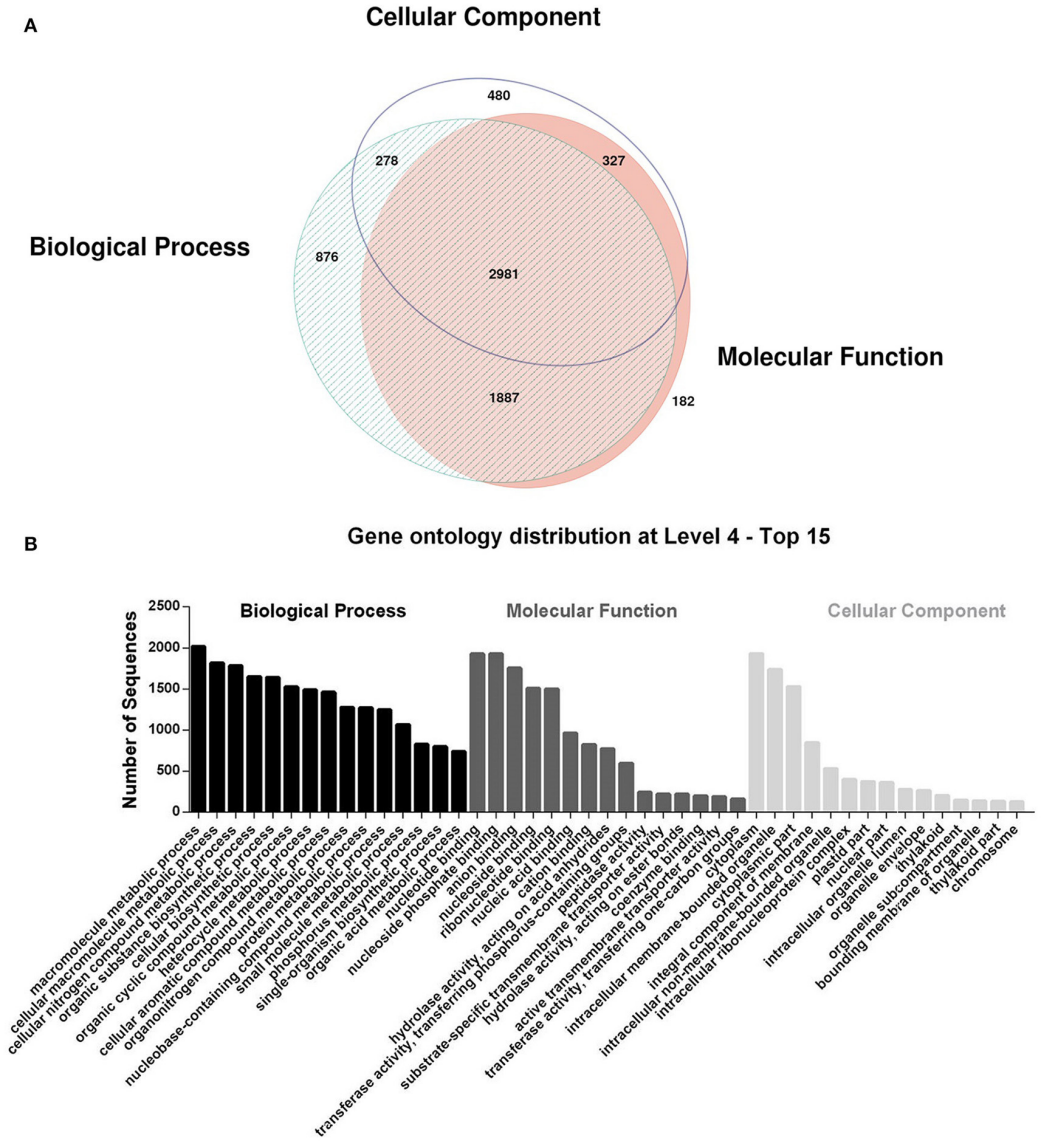
Gene Ontology annotation: **(A)** Venn diagram of the distribution of mapped contigs with Biological Process, Molecular Function and Cellular Component terms and **(B)** distribution of the Top 15 terms at level 4 in the three main categories. The intersection areas indicate the contigs mapped with terms from two or three categories. Venn diagram are performed by software eulerAPE v.3.0.

The search of these contigs against the NCBI protein non-redundant database with BLASTX demonstrates that 8,980 (52.19%) sequences had at least one hit. The mapping of the sequences against the GO database retrieved 7,009 sequences mapped, and all assigned GO terms were classified into three main categories: cellular component, molecular function, and biological process. The distribution of sequences mapped to the three different categories and the top 15 GO terms are represented in Figures 1A,B, respectively.

Thus, taking the comparative numbers of contigs, mean length, the clustering process and sequencing depth and coverage into account its likely that our assembly comprises a representative number of transcripts but which were partially reconstructed. Moreover, it is important to note that although we have used stringent blast parameters to remove contaminations, some contigs can still come from contamination. Therefore, experimental validation to confirm *P. crispa* origin of some contigs is warranted.

### Data validation and quality control

The reading quality of the data of this transcriptomic analysis was evaluated through FastQC software (Babraham Bioinformatics) [RRID:SCR_014583]. The paired-end reads results were merged using MultiQC (http://multiqc.info) and are shown in Supplementary Figure S1. Per base quality phred scores range from 32.78 to 40.06, indicating base call accuracies of >99.9% (Supplementary Figure S1A). Per sequence quality shows that 99.62% of reads had a mean phred score of 30 or above (Supplementary Figure S1B) and per base N content was low, with a maximum value 0.18% (Supplementary Figure S1C).

### Re-use potential

Through the data of this transcriptome, it is possible to perform searches for genes, aiming the heterologous expression of proteins with biotechnological potential, such as antifreeze proteins, which act to inhibit freezing of intracellular fluids (Nath et al., 2013), heat-shock proteins that play an important role in maintain biological activities in algae present in these acclimatization process (Li and Brawley, 2004) and mycosporine-like amino acids responsive to high incidence of ultraviolet radiation (Kováčik and Pereira, 2001). Proteomics approaches may also be employed, aiming at the confirmation of gene expression at the translational level.

## Author contributions

EC, LM, GW, PP: Conducted the experiment; EC, LM, PM, FD, MA, GW: Performed analysis on the data; FV, AP, JB, PP: Conceived the project and acquired funding; EC, LM, PM, GW, PP: Wrote the manuscript.

### Conflict of interest statement

The authors declare that the research was conducted in the absence of any commercial or financial relationships that could be construed as a potential conflict of interest.
