# Supplementary material for: De novo Assembly and Annotation of the Antarctic Alga Prasiola crispa Transcriptome
Source: Front Mol Biosci. 2018 Jan 8;4:89. doi: 10.3389/fmolb.2017.00089 (PMC5766667; doi:10.3389/fmolb.2017.00089)
Supplement: Supplementary file 1 [file Table2.PDF]

Supplementary Material

Article Title

Evelise Leis Carvalho[1#], Lucas Ferreira Maciel[1#], Pablo Echeverria Macedo[1], Filipe Zimmer Dezordi[1], Maria Eduarda Tabarez de Abreu[1], Filipe de Carvalho Victória[2], Antônio Batista Pereira[2], Juliano Tomazzoni Boldo[1], Gabriel da Luz Wallau[3], Paulo Marcos Pinto[1*]

# indicates equal contribution

*** Correspondence:**

Corresponding Author: paulopinto@unipampa.edu.br

1 Supplementary Figures and Tables

1.1 Supplementary Tables

Supplementary Table S1

Comparison between *Prasiola crispa* and organisms from the Trebouxiophyceae class with transcriptome sequenced.

**Attributes**	***Prasiola crispa***	***Chlorella minutissima***	***Trebouxia gelatinosa***	***Coccomyxa subellipsoidea***	***Chlorella sorokiniana***	***Botryococcus braunii***
**Total raw reads**	42,978,976	69,011,712	243,763,578	-	244,291,069	-
**Total processed reads**	5,233,428	67,559,338	237,404,631	46,000,000	229,228,757	-
**Number of contigs**	17,201	14,905	19,601	9,409	63,811	61,220
**Mean length**	763.1	2,998.04	1,605	-	1,022	-
**Sequences with at least one blast hit (%)**	52.19	53.50	53.60	-	36.80	-
**Largest contig (pb)**	12,802	-	31,749	-	15,932	-
**N50**	1,036	-	3,594	-	2,502	-
**Assembler**	Trinity	Trinity	Trinity	Reference genome	Trinity	Trinity

(-) Data not reported.

frontiers
