# Supplementary material for: De novo Assembly and Annotation of the Antarctic Alga Prasiola crispa Transcriptome
Source: Front Mol Biosci. 2018 Jan 8;4:89. doi: 10.3389/fmolb.2017.00089 (PMC5766667; doi:10.3389/fmolb.2017.00089)
Supplement: Supplementary file 2 [file Image1.PDF]

frontiers

Supplementary Material

Article Title

Evelise Leis Carvalho[1#], Lucas Ferreira Maciel[1#], Pablo Echeverria Macedo[1], Filipe Zimmer Dezordi[1], Maria Eduarda Tabarez de Abreu[1], Filipe de Carvalho Victória[2], Antônio Batista Pereira[2], Juliano Tomazzoni Boldo[1], Gabriel da Luz Wallau[3], Paulo Marcos Pinto[1*]

# indicates equal contribution

*** Correspondence:**

Corresponding Author: paulopinto@unipampa.edu.br

1 Supplementary Figures and Tables

1.1 Supplementary Figures

A

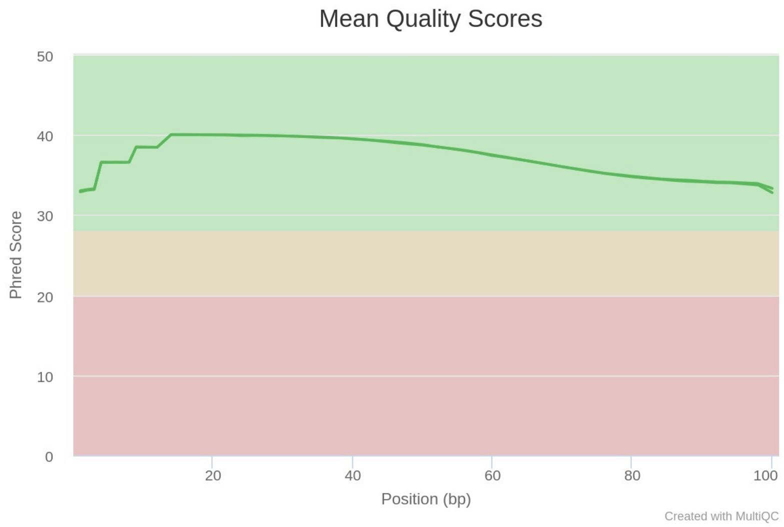
Mean Quality Scores
Phred Score
Position (bp)
Created with MultiQC

B

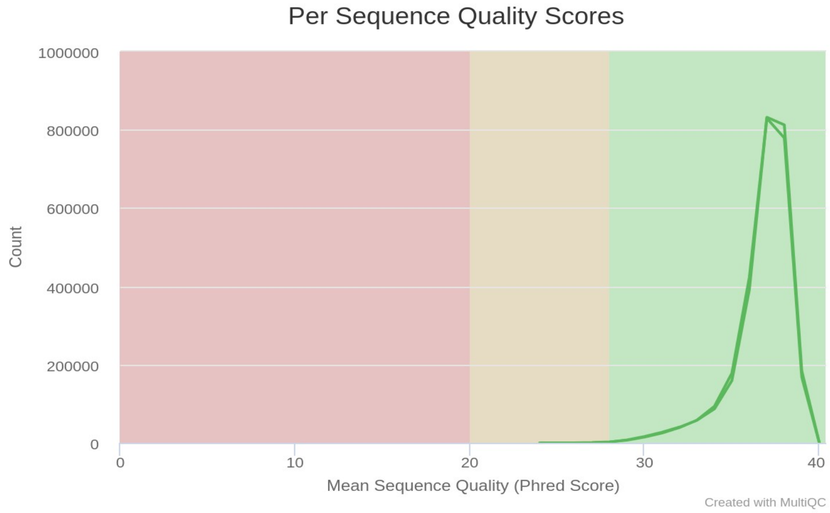
Per Sequence Quality Scores
Count
Mean Sequence Quality (Phred Score)
Created with MultiQC

C

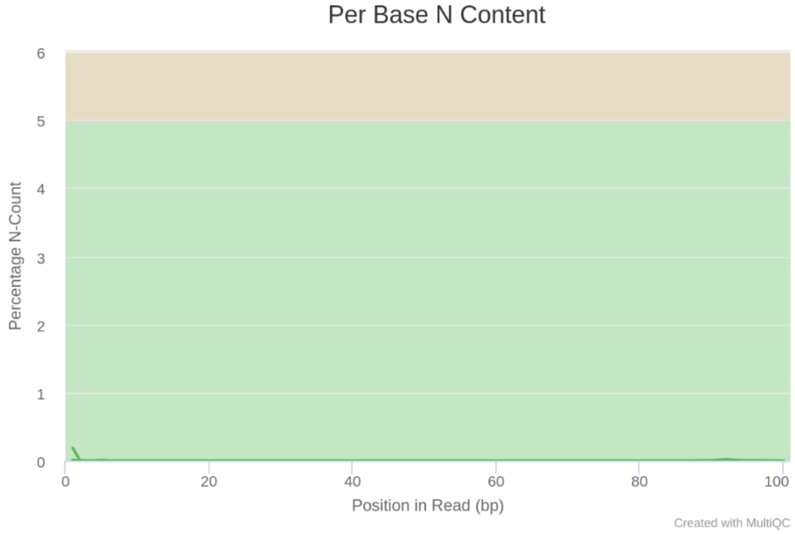
Per Base N Content
Percentage N-Count
Position in Read (bp)
Created with MultiQC

Supplementary Figure S1. Graphs about sequencing read quality generated with FastQC. (A) Per base quality phred. (B) Per sequence quality. (C) Per base N content. The colored areas separate the metrics into High (green), Medium (yellow) and Low (red) quality.
